# Supplementary figures and images for: Network pharmacology predicts combinational effect of novel herbal pair consist of Ephedrae herba and Coicis semen on adipogenesis in 3T3-L1 cells
Source: PLoS One. 2023 Mar 16;18(3):e0282875. doi: 10.1371/journal.pone.0282875 (PMC10019655; doi:10.1371/journal.pone.0282875)

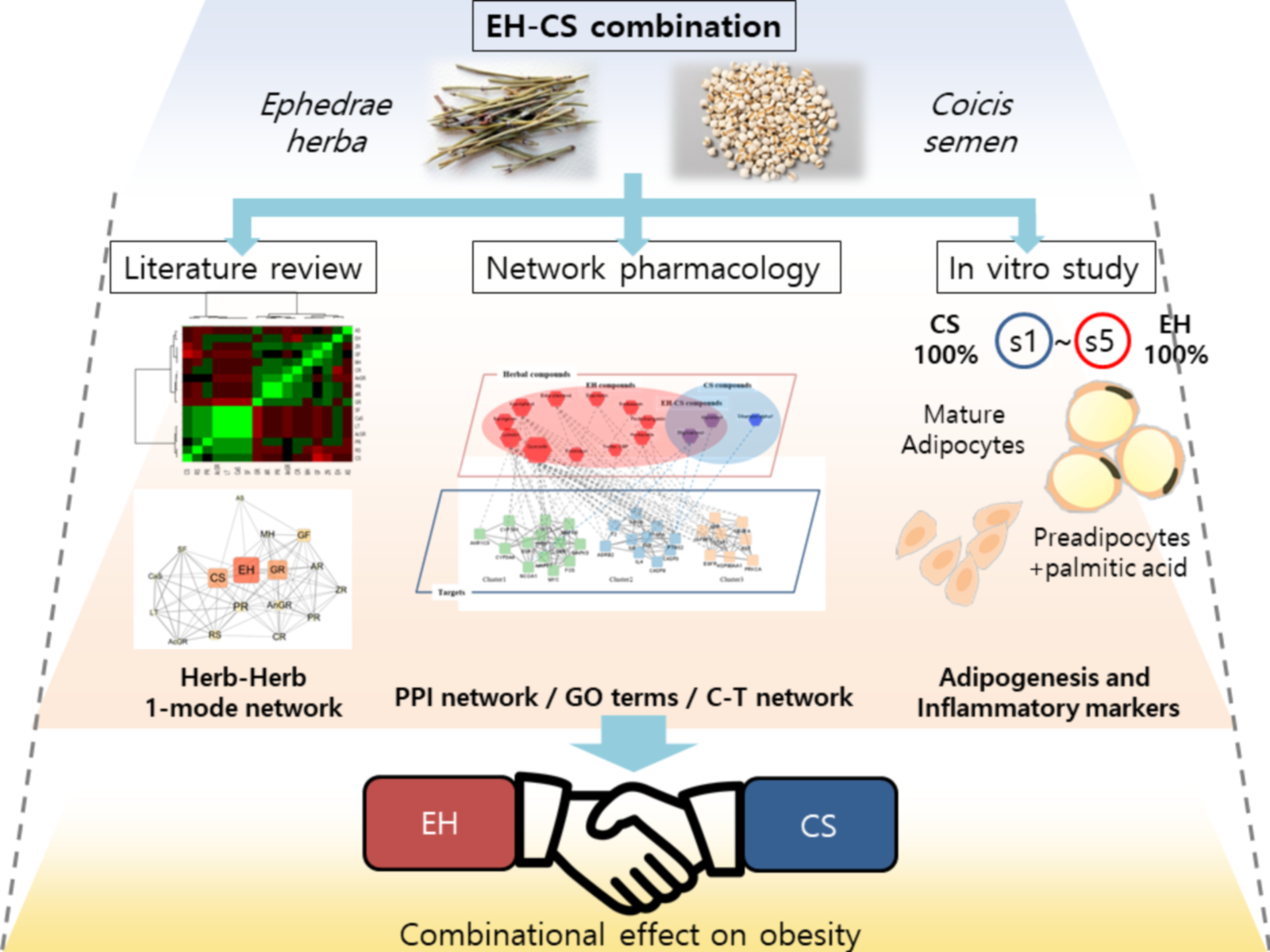

Supplement: S1 Graphical abstract — (PNG) [file pone.0282875.s001.png]
