# Supplementary material for: Network pharmacology predicts combinational effect of novel herbal pair consist of Ephedrae herba and Coicis semen on adipogenesis in 3T3-L1 cells
Source: PLoS One. 2023 Mar 16;18(3):e0282875. doi: 10.1371/journal.pone.0282875 (PMC10019655; doi:10.1371/journal.pone.0282875)
Supplement: S1 Raw images — (PDF) [file pone.0282875.s002.pdf]

Western blot image

Network pharmacology predicts combinational effect of novel herbal pair consist of Ephedrae herba and Coicis semen on adipogenesis in 3T3-L1 cells

COX-2 Western blot

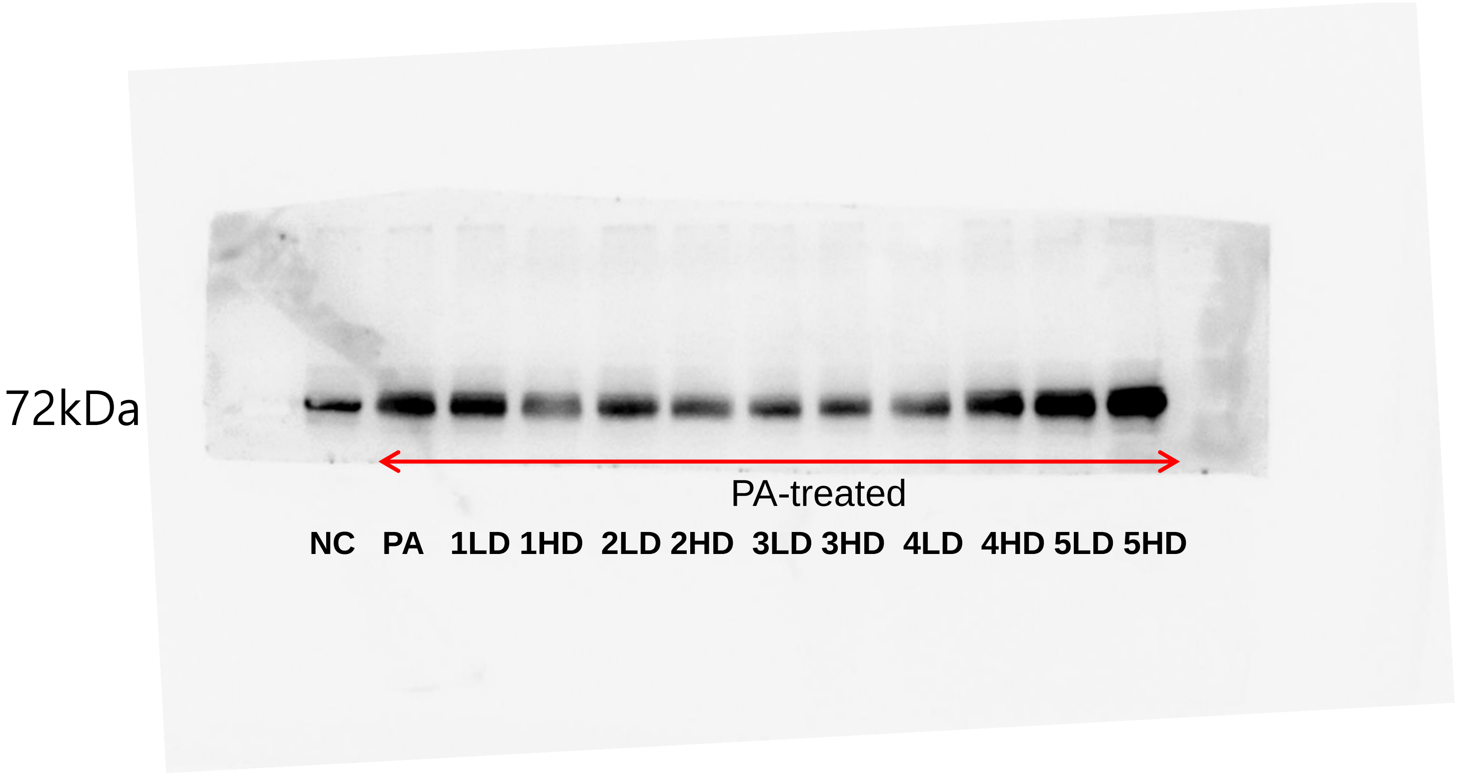

PA: palmitic acid-treated
1~5 : EH-CS combination sample
LD : 10 μg/ml, HD : 25 μg/ml

COX-2 Western blot

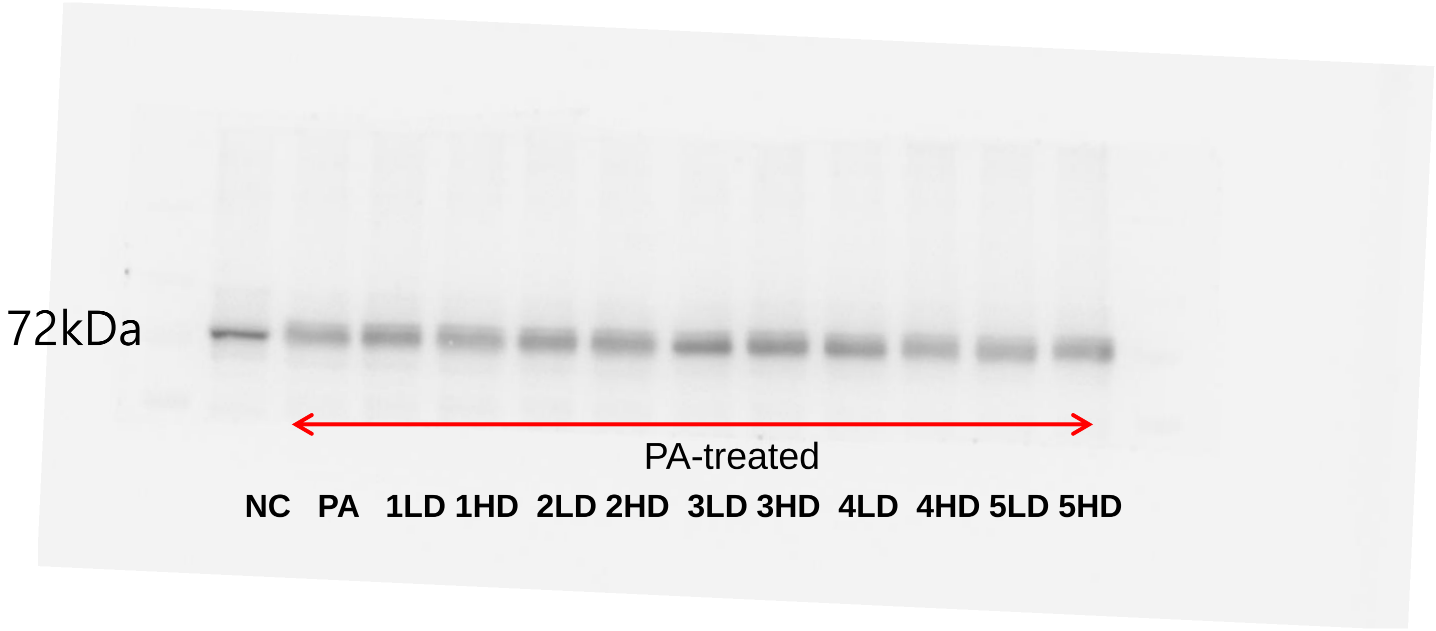

PA: palmitic acid-treated
1~5 : EH-CS combination sample
LD : 10 μg/ml, HD : 25 μg/ml

COX-2 Western blot

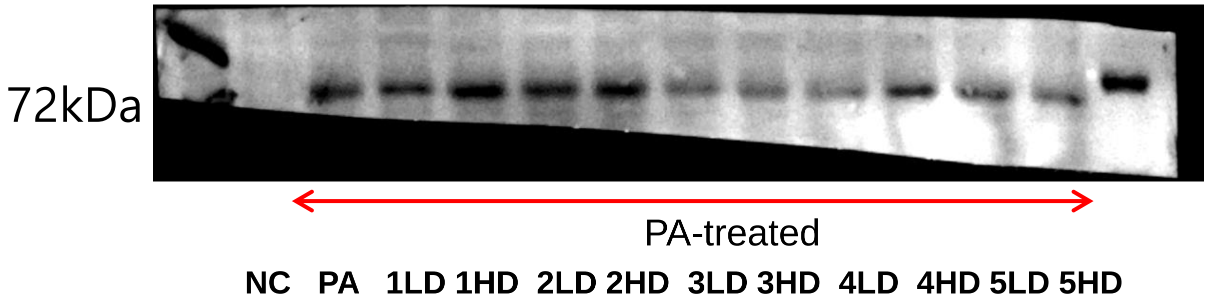

PA: palmitic acid-treated
1~5 : EH-CS combination sample
LD : 10 μg/ml, HD : 25 μg/ml

beta actin for COX-2

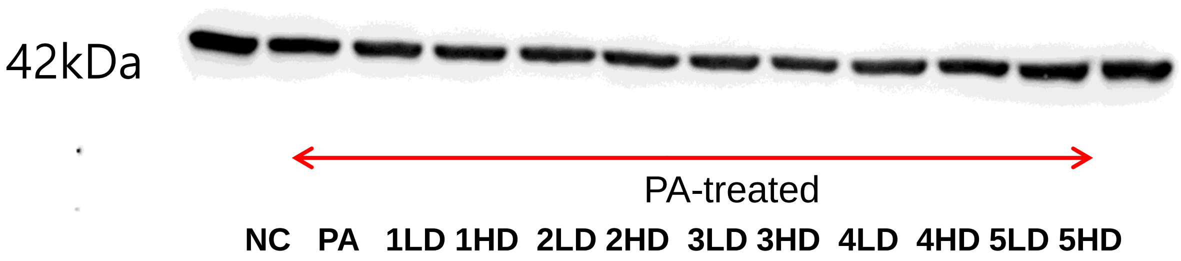

PA: palmitic acid-treated
1~5 : EH-CS combination sample
LD : 10 μg/ml, HD : 25 μg/ml

beta actin for COX-2

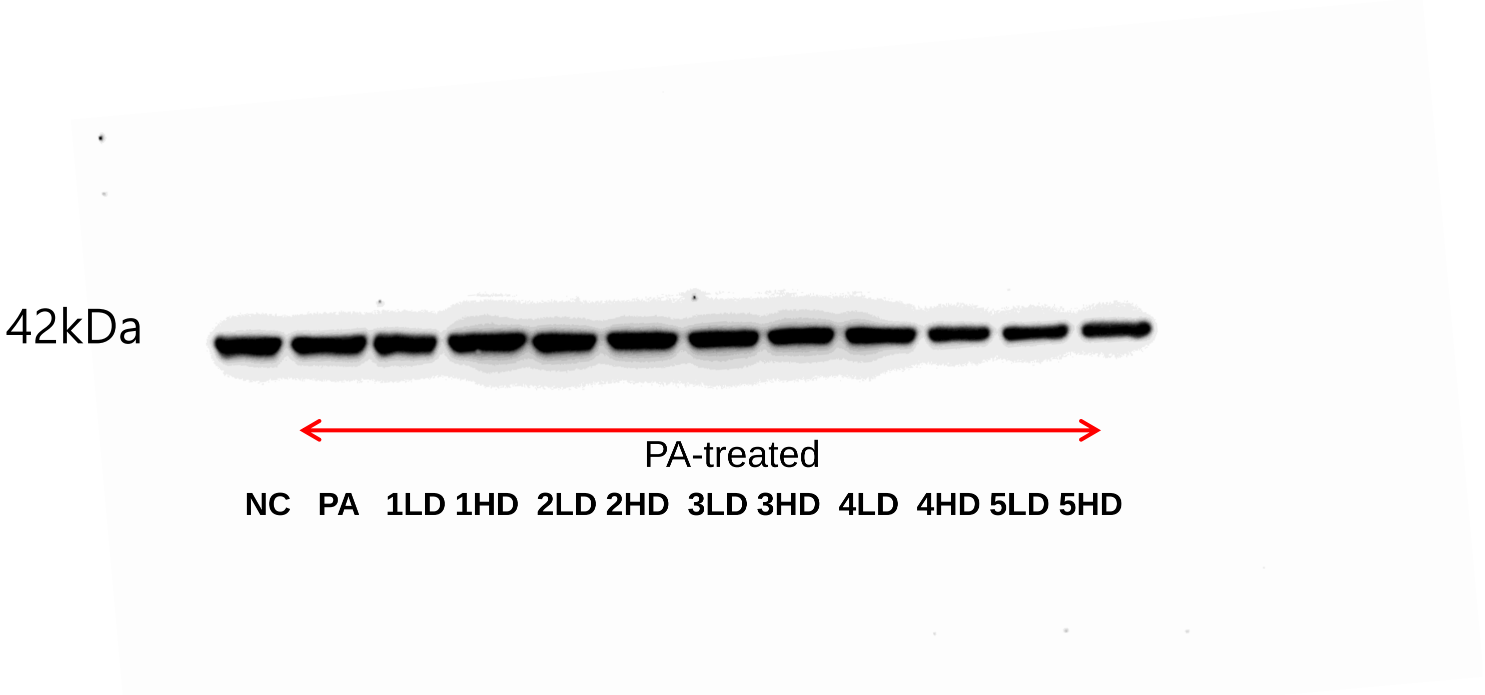

PA: palmitic acid-treated
1~5 : EH-CS combination sample
LD : 10 μg/ml, HD : 25 μg/ml

beta actin for COX-2

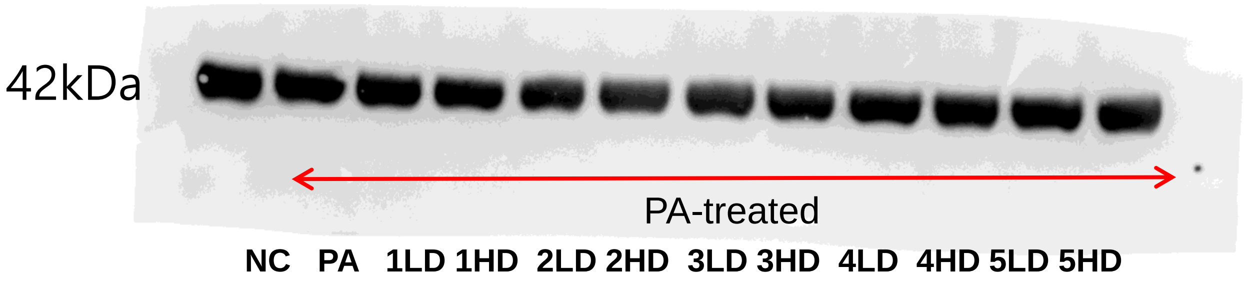

PA: palmitic acid-treated
1~5 : EH-CS combination sample
LD : 10 μg/ml, HD : 25 μg/ml

P-AMPK

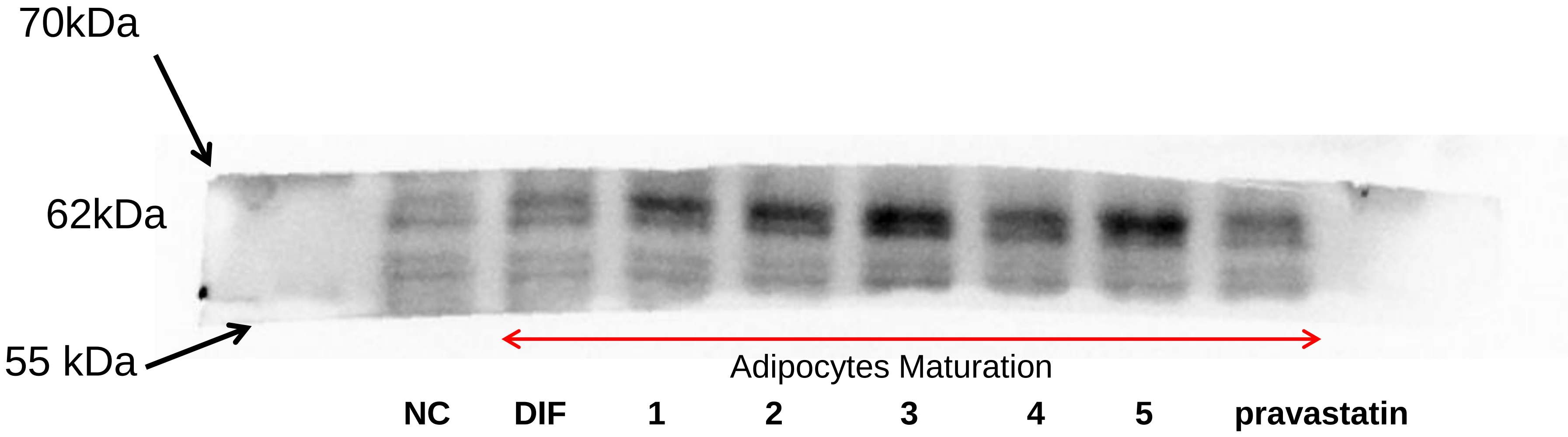

DIF: differentiated adipocytes
1~5 : EH-CS combination sample
All 25 μg/ml (pravastatin 50 μg/ml)

P-AMPK

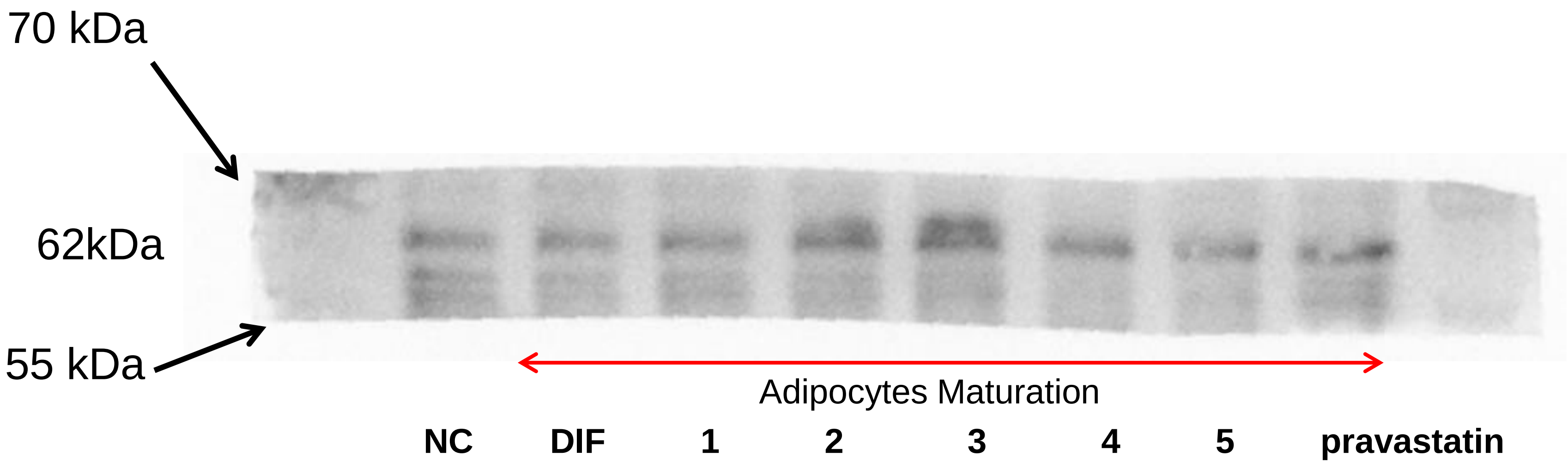

DIF: differentiated adipocytes
1~5 : EH-CS combination sample
All 25 μg/ml (pravastatin 50 μg/ml)

P-AMPK

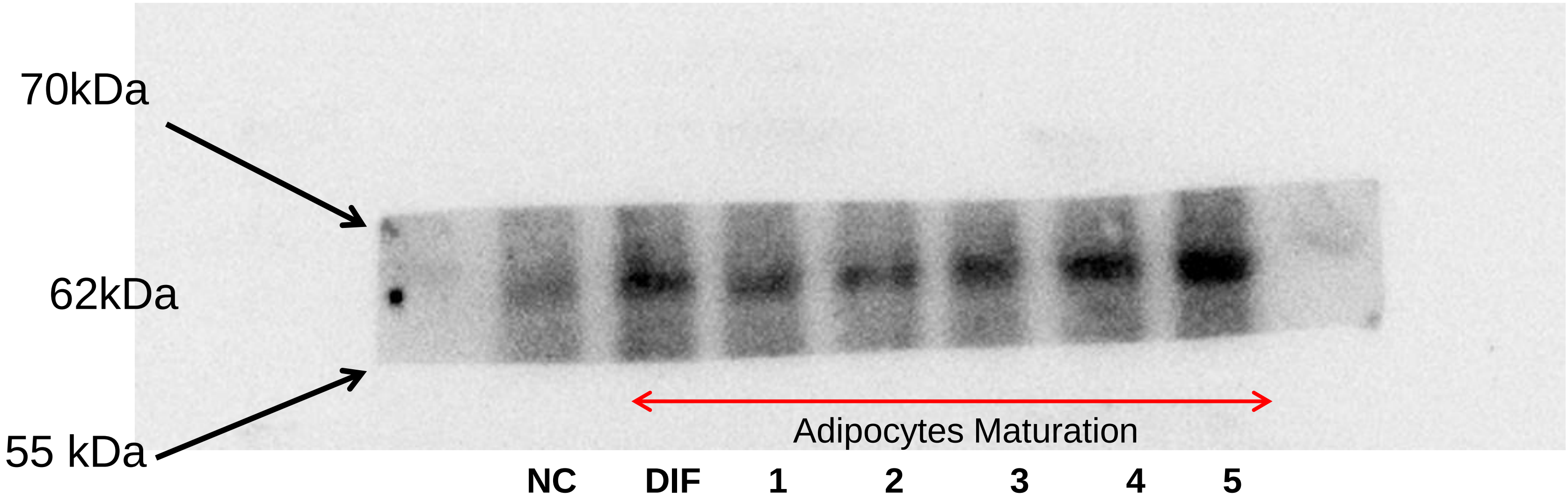

DIF: differentiated adipocytes
1~5 : EH-CS combination sample
All 25 μg/ml

P-AMPK

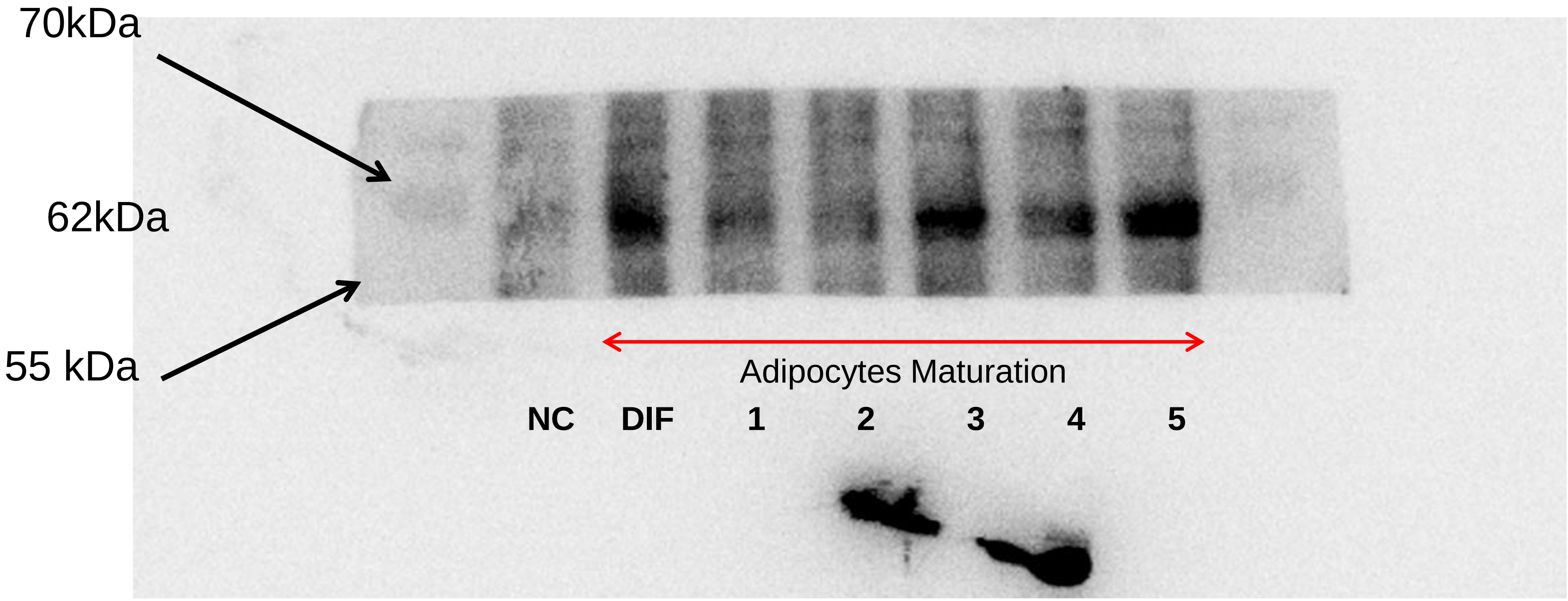

DIF: differentiated adipocytes
1~5 : EH-CS combination sample
All 25 μg/ml

AMPK

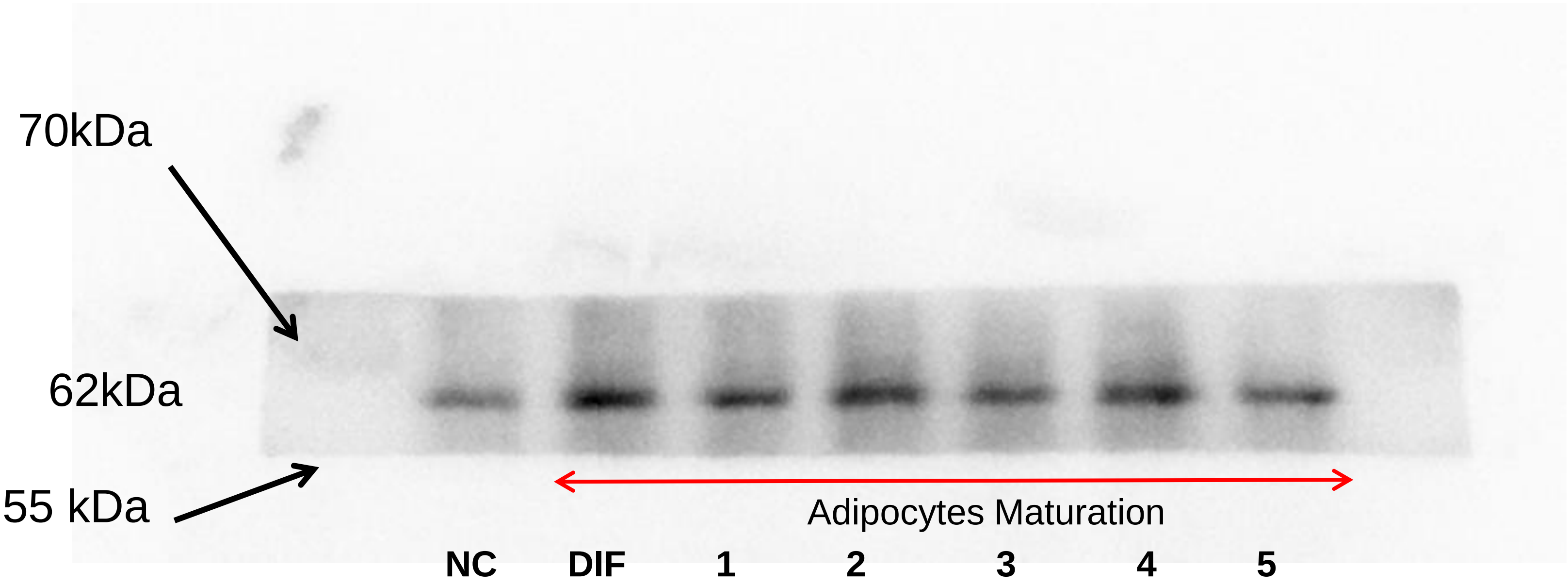

DIF: differentiated adipocytes
1~5 : EH-CS combination sample
All 25 μg/ml

AMPK

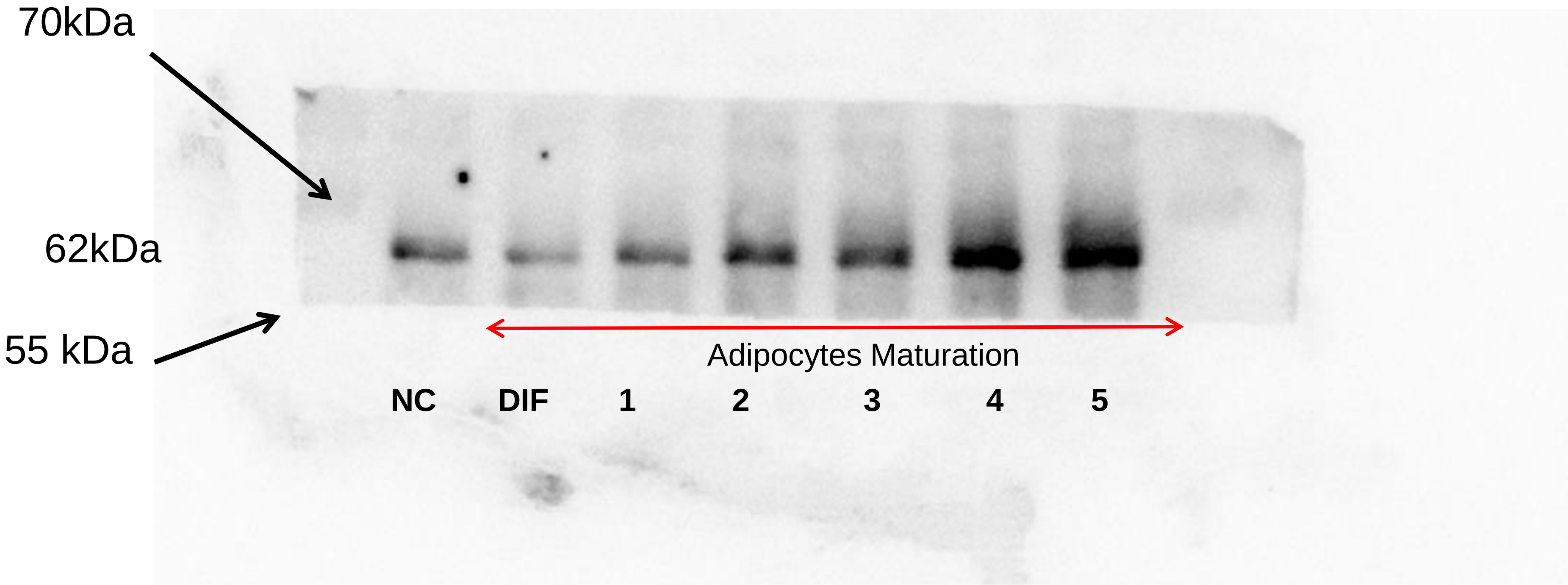

DIF: differentiated adipocytes
1~5 : EH-CS combination sample
All 25 μg/ml

AMPK

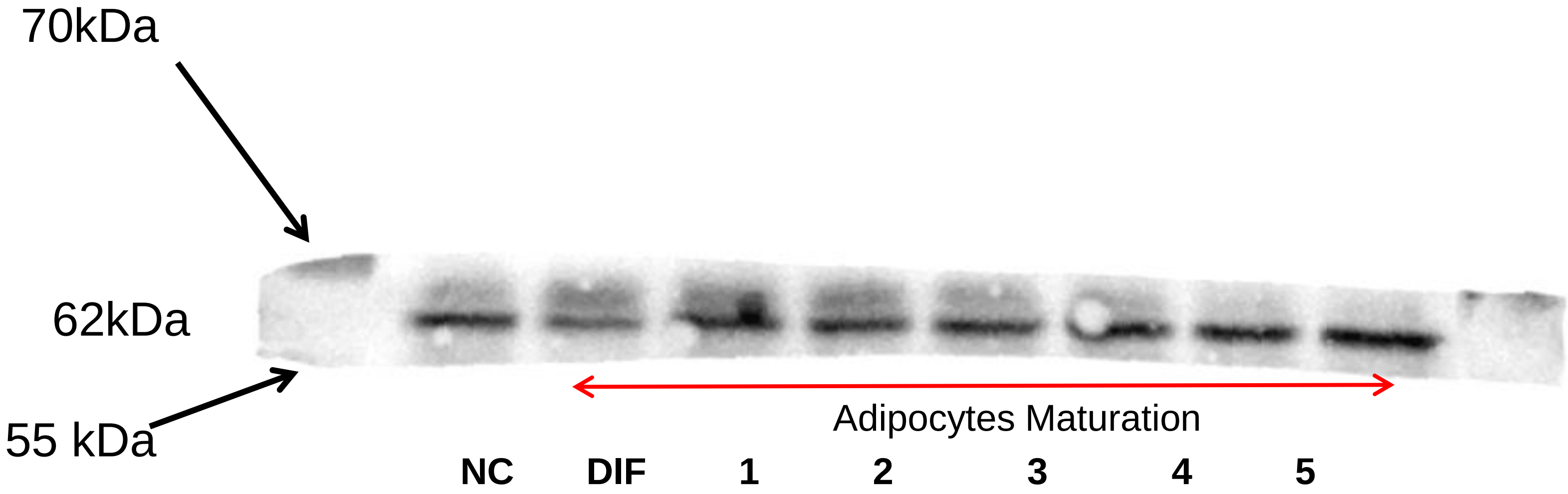

DIF: differentiated adipocytes
1~5 : EH-CS combination sample
All 25 μg/ml

beta actin for AMPK

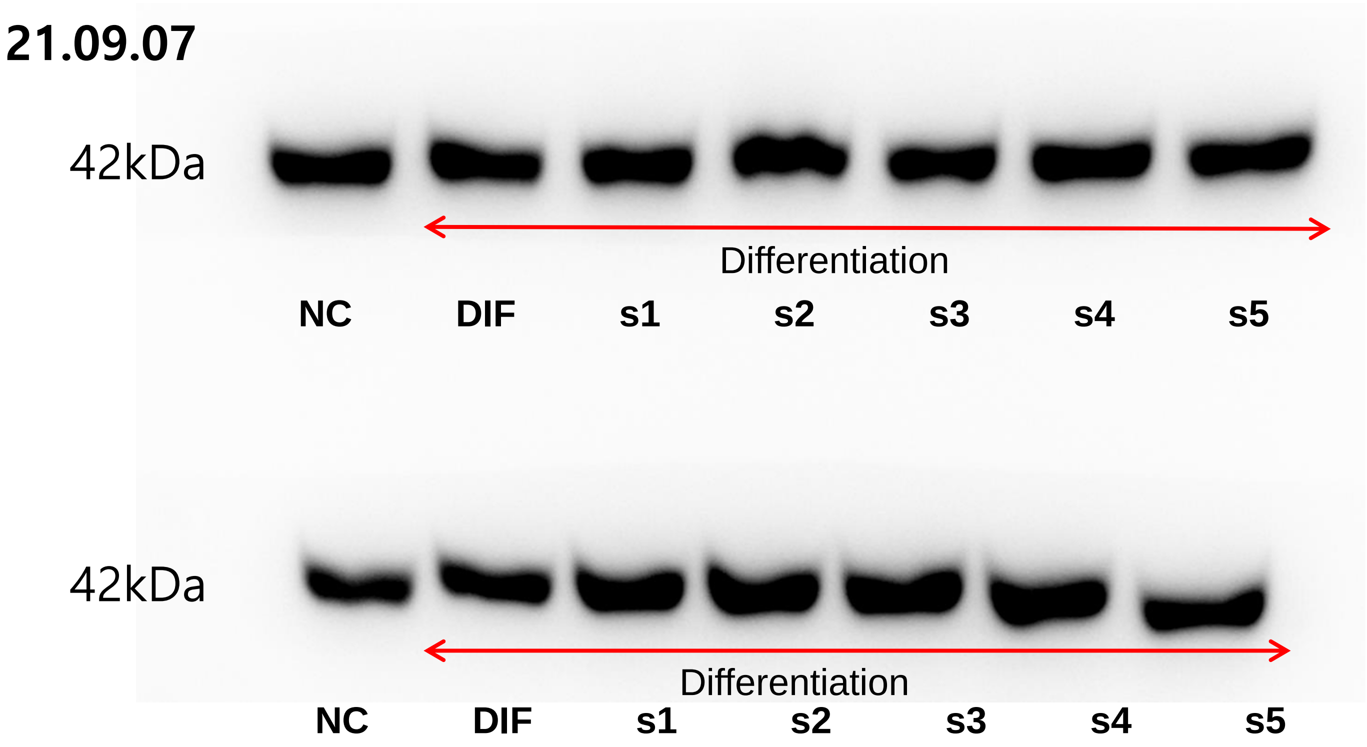

DIF: differentiated adipocytes
1~5 : EH-CS combination sample
All 25 μg/ml

beta actin for AMPK

21.10.26

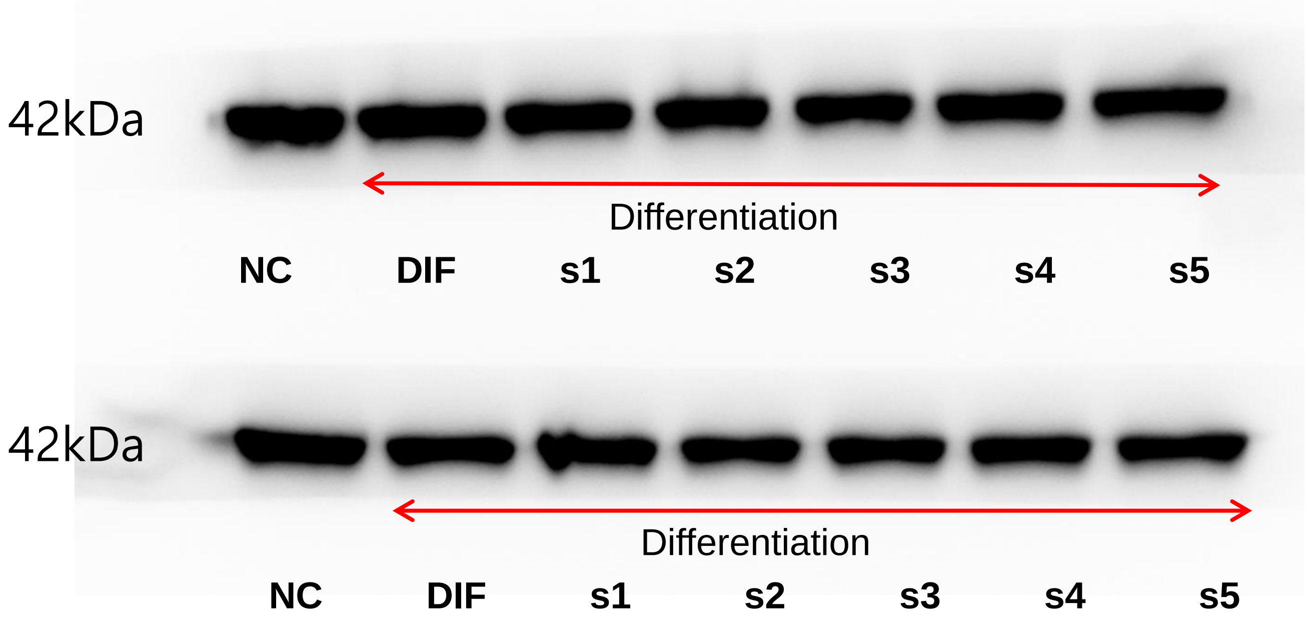

DIF: differentiated adipocytes
1~5 : EH-CS combination sample
All 25 μg/ml
